# SMAD3 and SMAD4 have a more dominant role than SMAD2 in TGFβ-induced chondrogenic differentiation of bone marrow-derived mesenchymal stem cells

**DOI:** 10.1038/srep43164

**Published:** 2017-02-27

**Authors:** Laurie M. G. de Kroon, Roberto Narcisi, Guus G. H. van den Akker, Elly L. Vitters, Esmeralda N. Blaney Davidson, Gerjo J. V. M. van Osch, Peter M. van der Kraan

**Affiliations:** 1 Experimental Rheumatology, Department of Rheumatology, Radboud University Medical Center, Nijmegen, 6500 HB, The Netherlands; 2 Department of Orthopedics, Erasmus MC University Medical Center, Rotterdam, 3015 CN, The Netherlands; 3 Department of Otorhinolaryngology, Erasmus MC University Medical Center, Rotterdam, 3015 CN, The Netherlands

## Abstract

To improve cartilage formation by bone marrow-derived mesenchymal stem cells (BMSCs), the signaling mechanism governing chondrogenic differentiation requires better understanding. We previously showed that the transforming growth factor-β (TGFβ) receptor ALK5 is crucial for chondrogenesis induced by TGFβ. ALK5 phosphorylates SMAD2 and SMAD3 proteins, which then form complexes with SMAD4 to regulate gene transcription. By modulating the expression of SMAD2, SMAD3 and SMAD4 in human BMSCs, we investigated their role in TGFβ-induced chondrogenesis. Activation of TGFβ signaling, represented by SMAD2 phosphorylation, was decreased by SMAD2 knockdown and highly increased by SMAD2 overexpression. Moreover, TGFβ signaling via the alternative SMAD1/5/9 pathway was strongly decreased by SMAD4 knockdown. TGFβ-induced chondrogenesis of human BMSCs was strongly inhibited by SMAD4 knockdown and only mildly inhibited by SMAD2 knockdown. Remarkably, both knockdown and overexpression of SMAD3 blocked chondrogenic differentiation. Chondrogenesis appears to rely on a delicate balance in the amount of SMAD3 and SMAD4 as it was not enhanced by SMAD4 overexpression and was inhibited by SMAD3 overexpression. Furthermore, this study reveals that TGFβ-activated phosphorylation of SMAD2 and SMAD1/5/9 depends on the abundance of SMAD4. Overall, our findings suggest a more dominant role for SMAD3 and SMAD4 than SMAD2 in TGFβ-induced chondrogenesis of human BMSCs.

Joint injuries frequently cause articular cartilage lesions that do not heal well in adults as articular cartilage has poor regenerative capacity. Since bone marrow-derived mesenchymal stem cells (BMSCs) can chondrogenically differentiate, they are promising for cell-based regeneration of damaged articular cartilage. Chondrogenic differentiation is potently induced by transforming growth factor-β (TGFβ)^1^,^2^,^3^. However, cartilage tissue formed by BMSC-derived chondrocytes does not completely resemble native articular cartilage as these cells tend to lose the chondrogenic phenotype due to hypertrophic differentiation^4^,^5^,^6^,^7^,^8^. Therefore, understanding how mediators of TGFβ signal transduction govern chondrogenesis will be crucial to improve cartilage regeneration by BMSCs.

Binding of TGFβ to its type II serine/threonine receptor TGFBR2 activates the type I receptor TGFBR1, also termed activin receptor-like kinase 5 (ALK5), to intracellularly phosphorylate receptor-regulated SMAD proteins (R-SMADs). Activated R-SMADs form complexes with co-factor SMAD4 and these complexes translocate to the nucleus where they regulate gene transcription^9^. Previously, we demonstrated that either blocking the kinase activity or downregulating the expression of ALK5 in human BMSCs inhibits chondrogenesis^6^,^10^, indicating an important role for TGFβ signaling via ALK5 in chondrogenic differentiation of BMSCs.

ALK5 can activate two R-SMADs, SMAD2 and SMAD3, which share similar structures including a Mad-Homology 1 (MH1) domain, linker region and MH2 domain^9^,^11^. Despite their similarities, SMAD2 and SMAD3 have a differential role in TGFβ signal transduction^12^,^13^,^14^,^15^. Whereas *Smad2* knockout mice die during gastrulation^16^,^17^, *Smad3* knockout mice develop cartilage and survive for several months after birth^18^,^19^,^20^. Moreover, SMAD3, but not SMAD2, is involved in enhanced transcriptional activity of *SRY (Sex Determining Region Y)-Box 9 (SOX9)*; a master regulator of chondrogenesis, in human mesenchymal stem cells^21^,^22^. In contrast to SMAD3, SMAD2 lacks the MH1 domain. Therefore, SMAD2 cannot bind DNA without complex formation with SMAD4, which may explain the differential effects of SMAD2 and SMAD3^23^,^24^,^25^,^26^,^27^. However, whether SMAD2 and SMAD3 have a different function during TGFβ-induced chondrogenic differentiation of human BMSCs remains largely unknown. Also the specific role of SMAD4 in chondrogenesis of human BMSCs has not been investigated. In mice, deletion of *Smad4* causes early embryonic death^28^,^29^. Therefore, tissue-specific *Smad4* knockout mice have been generated. Conditional deletion of *Smad4* in the limb bud mesenchyme of mice leads to an absence of cartilage elements prefiguring the limb skeleton^30^,^31^, indicating that SMAD4 is required for cartilage formation. Furthermore, chondrocyte-specific *Smad4* knockout mice exhibit dwarfism and impaired growth plate organization^32^. Similarly, *Smad3* knockout mice have forelimb malformations, are smaller than wild-type mice and they develop spontaneous joint degeneration resembling the degenerative joint disease osteoarthritis in humans^18^,^19^. In humans, mutations in *SMAD3* lead to skeletal anomalies and early-onset of osteoarthritis^33^,^34^,^35^,^36^. Moreover, SMAD3 is required for TGFβ-mediated suppression of hypertrophic differentiation of human articular chondrocytes^37^,^38^. Although SMAD3 does not appear directly required for embryonic cartilage and joint development, SMAD3 seems to be important for maintaining a stable cartilage phenotype by preventing cartilage degeneration.

Since SMAD2, SMAD3 and SMAD4 have been shown to differentially regulate TGFβ signaling and to have distinct functions during *in vivo* cartilage development and maintenance, we investigated their role in TGFβ-induced signaling and chondrogenesis of human BMSCs. We knocked down and overexpressed either SMAD2, SMAD3 or SMAD4 in human fetal BMSCs and determined the effects on TGFβ-activated SMAD phosphorylation and induction of chondrogenic differentiation.

## Results

### Efficient knockdown and overexpression of SMAD2, SMAD3 and SMAD4

To study the role of SMAD2, SMAD3 and SMAD4 in TGFβ-induced SMAD phosphorylation and chondrogenesis of human BMSCs, their expression was modulated in human fetal BMSCs using short hairpin RNA (shRNA)-mediated knockdown and adenoviral (ad)-mediated overexpression.

**Figure 1:**
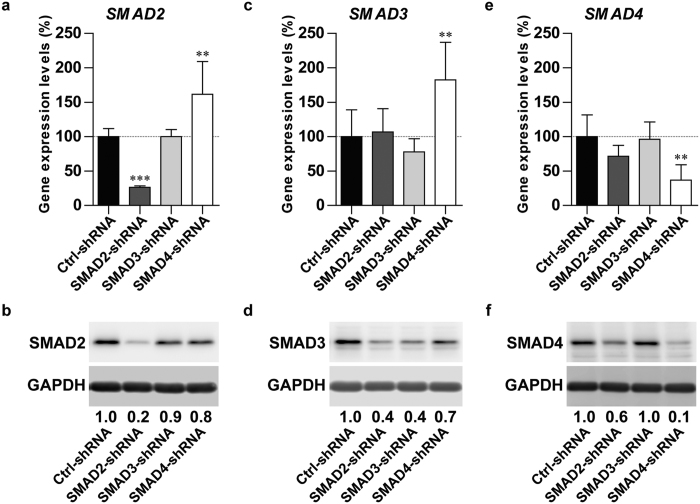
Short hairpin RNA-mediated knockdown of SMAD2, SMAD3 and SMAD4 expression. Following lentiviral transduction with either SMAD2-shRNA, SMAD3-shRNA, SMAD4-shRNA or non-mammalian shRNA control (Ctrl-shRNA), human fetal BMSCs were cultured in chondrogenic medium for 1 day. Short hairpin RNA-mediated knockdown was evaluated by determining gene (RT-qPCR; (**a**,**c**,**e**) and protein (Western blot; (**b**,**d**,**f**) expression of SMAD2 (**a**,**b**), SMAD3 (**c**,**d**) and SMAD4 (**e**,**f**). Protein levels were normalized to GAPDH and expressed as relative to Ctrl-shRNA. Gene expression was normalized to the mean C_T_ value of *RPS27a* and *TBP*. Data are expressed as % relative to normalized mRNA levels in Ctrl-shRNA. Bars represent mean + S.D. of triplicate pellets from 2 experiments. **p < 0.01; ***p < 0.001 compared to Ctrl-shRNA.

**Figure 2:**
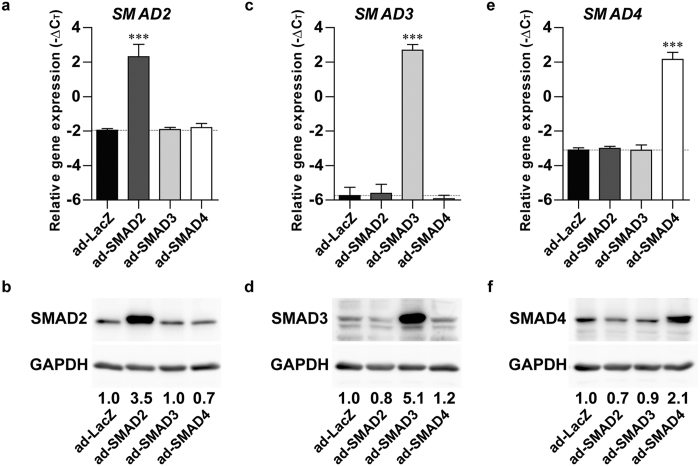
Adenoviral-mediated overexpression of SMAD2, SMAD3 and SMAD4. Human fetal BMSCs were transduced with adenovirus overexpressing SMAD2 (ad-SMAD2), ad-SMAD3, ad-SMAD4 or ad-LacZ as control, followed by culturing in chondrogenic medium for 1 day. To confirm overexpression, gene (RT-qPCR; (**a**,**c**,**e**) and protein (Western blot; (**b**,**d**,**f**) expression of SMAD2 (**a**,**b**), SMAD3 (**c**,**d**) and SMAD4 (**e**,**f**) were measured. Protein levels were normalized to GAPDH and expressed as relative to ad-LacZ. Gene expression data are presented as −ΔC_T_ compared to the mean C_T_ value of *RPS27a* and *TBP*. Bars represent mean + S.D. of triplicate pellets from 2 experiments. ***p < 0.001 compared to ad-LacZ.

We confirmed efficient shRNA-mediated knockdown of SMAD2 (Fig. 1a,b), SMAD3 (Fig. 1c,d) and SMAD4 (Fig. 1e,f) at the mRNA and protein level. In addition, we observed that SMAD2 (Fig. 1a) and SMAD3 (Fig. 1c) mRNA levels, but not their protein levels (Fig. 1b,d), were increased in SMAD4-shRNA compared to Ctrl-shRNA. Although gene expression of SMAD3 (Fig. 1c) and SMAD4 (Fig. 1e) was similar between SMAD2-shRNA and Ctrl-shRNA, their protein expression (Fig. 1d,f) was decreased in SMAD2-shRNA. Next, we verified adenoviral overexpression of SMAD2 (Fig. 2a,b), SMAD3 (Fig. 2c,d) and SMAD4 (Fig. 2e,f) at gene and protein level. Notably, only SMAD4 protein (Fig. 2f) was slightly reduced in ad-SMAD2 compared to ad-LacZ. Thus, these data confirm efficient knockdown and overexpression of SMAD2, SMAD3 and SMAD4.

### TGFβ-activated phosphorylation of R-SMADs is altered by knockdown and overexpression of SMAD2, SMAD3 or SMAD4

**Figure 3:**
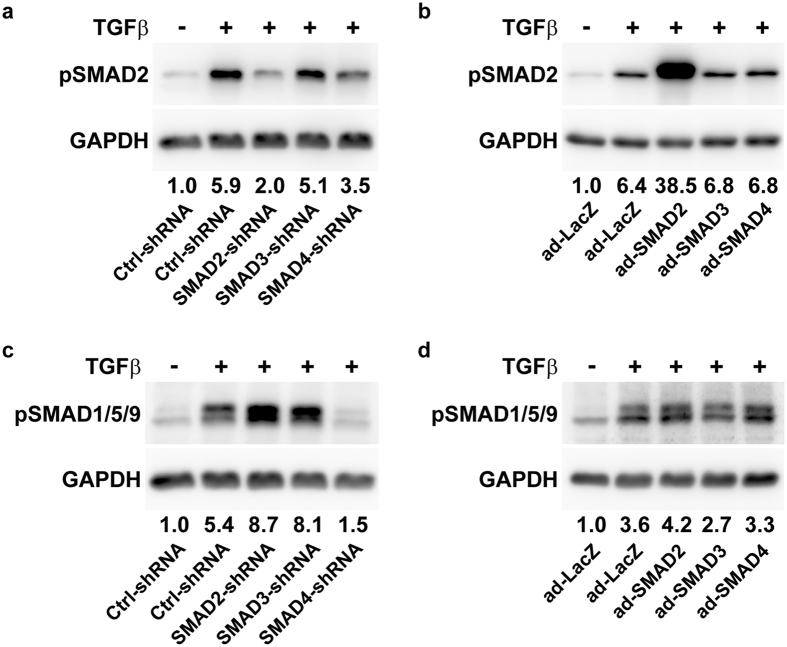
TGFβ-activated phosphorylation of R-SMADs is affected by knockdown and overexpression of SMAD2, SMAD3 and SMAD4. The effects of shRNA-mediated knockdown and adenoviral-mediated overexpression of SMAD2, SMAD3 and SMAD4 on TGFβ-induced phosphorylation of SMAD2 (pSMAD2; (**a**,**b**) and pSMAD1/5/9 (**c**,**d**) were determined using Western blot. To confirm TGFβ-induced phosphorylation of SMAD2 and SMAD1/5/9, Ctrl-shRNA cells and ad-LacZ cells were either not stimulated (−) or stimulated (+) with TGFβ in chondrogenic medium. Protein levels were normalized to GAPDH and expressed as relative to unstimulated Ctrl-shRNA or ad-LacZ.

TGFβ signaling appears to be a straightforward cascade in which the ALK5 receptor phosphorylates SMAD2 and SMAD3, which upon binding to SMAD4 translocate to the nucleus where they regulate gene transcription^9^. However, this system is more complex as multiple mechanisms have been discovered that control TGFβ signal transduction^39^. Since it remained unknown whether R-SMAD phosphorylation depends on the amount of SMAD2 and SMAD3 present, we investigated whether modulating the expression of SMAD2 and SMAD3 affected TGFβ-activated phosphorylation of R-SMADs. We only show the effects on phosphorylated SMAD2 (pSMAD2) due to difficult detection of pSMAD3. First, we verified that TGFβ induced pSMAD2 in Ctrl-shRNA cells (Fig. 3a) and in ad-LacZ cells compared to no stimulation (Fig. 3b). TGFβ-activated SMAD2 phosphorylation was reduced in the SMAD2-shRNA condition (Fig. 3a) and it was enhanced in ad-SMAD2 (Fig. 3b). No effect on pSMAD2 was observed with SMAD3-shRNA (Fig. 3a) or ad-SMAD3 (Fig. 3b). Moreover, to determine if co-factor SMAD4 is involved in TGFβ-activated R-SMAD phosphorylation, the expression of SMAD4 was modulated. We found that pSMAD2 was lower in SMAD4-shRNA than in Ctrl-shRNA (Fig. 3a) and was similar between ad-SMAD4 and ad-LacZ (Fig. 3b).

Next to SMAD2/3, TGFβ can activate SMAD1/5/9, and both these R-SMAD signaling pathways are important for chondrogenic induction^6^,^10^. Moreover, the TGFβ receptor ALK5 is required for TGFβ-activated phosphorylation of SMAD2/3 and SMAD1/5/9^40^. Hence, we hypothesized there could be competition between SMAD2/3 and SMAD1/5/9 for phosphorylation by ALK5. We confirmed SMAD1/5/9 phosphorylation (pSMAD1/5/9) in response to TGFβ stimulation compared to no stimulation in Ctrl-shRNA (Fig. 3c) and ad-LacZ (Fig. 3d). TGFβ-induced pSMAD1/5/9 was higher in SMAD2-shRNA and SMAD3-shRNA than in Ctrl-shRNA (Fig. 3c). Although SMAD1/5/9 phosphorylation was similar between ad-SMAD2 and ad-LacZ, it was slightly reduced in ad-SMAD3 (Fig. 3d). Next, we studied the involvement of SMAD4 in TGFβ-activated SMAD1/5/9 phosphorylation. We observed that in the SMAD4-shRNA condition TGFβ stimulation did not lead to induction of pSMAD1/5/9 (Fig. 3c), whereas TGFβ-induced pSMAD1/5/9 was comparable between ad-SMAD4 and ad-LacZ (Fig. 3d).

Taken together, these data demonstrate that activation of the SMAD2/3 and SMAD1/5/9 signaling pathways by TGFβ was affected by modulating SMAD2, SMAD3 or SMAD4 expression, indicating that the levels of TGFβ-induced phosphorylated R-SMADs depend on the amount of R-SMAD2/3 and co-SMAD4.

### Knocking down either SMAD3 or SMAD4 strongly inhibits chondrogenesis

We determined the contribution of SMAD2, SMAD3 and SMAD4 during TGFβ-induced chondrogenic differentiation by knocking down the expression of these SMADs and culturing the BMSCs in pellets in chondrogenic medium containing TGFβ. After 1, 7 and 14 days we evaluated expression of chondrogenesis-specific genes *ACAN, COL2A1* and *SOX9*, formation of cartilage matrix and the macroscopic appearance of the BMSC pellets.

**Figure 4:**
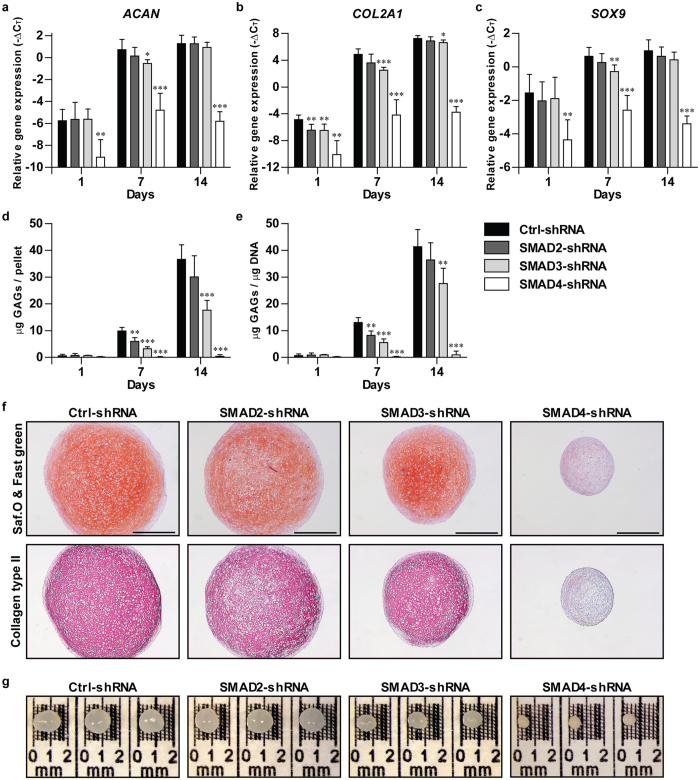
Chondrogenesis is mildly inhibited by SMAD2-shRNA, is strongly inhibited by SMAD3-shRNA and completely blocked by SMAD4-shRNA. Human fetal BMSCs were transduced (lentivirus) either with SMAD2-shRNA, SMAD3-shRNA, SMAD4-shRNA or Ctrl-shRNA and subsequently pellet-cultured for 1, 7 or 14 days in chondrogenic medium with TGFβ. The effect of shRNA-mediated knockdown of SMAD2, SMAD3 and SMAD4 on chondrogenesis was determined by RT-qPCR analysis of the chondrogenesis-specific genes; *ACAN* (**a**), *COL2A1* (**b**) and *SOX9* (**c**), deposition of glycosaminoglycans (GAGs) per pellet (**d**) and per μg DNA (**e**), histological examination of GAGs by Safranin O (Saf.O) staining and collagen type II by immunohistochemistry in sections of 14 days-cultured pellets (**f**), and macroscopic evaluation of 14 days-cultured pellets (**g**). In (**f**) representative images of consecutive pellet sections per condition are shown and the scale bar represents 500 μm. Gene expression data are presented as −ΔC_T_ compared to the mean C_T_ value of *RPS27a* and *TBP*. Bars represent mean + S.D. of triplicate pellets from 2 experiments. *p < 0.05; **p < 0.01; ***p < 0.001 compared to Ctrl-shRNA.

In 1, 7 and 14 days-cultured BMSC pellets, *ACAN* (Fig. 4a), *COL2A1* (Fig. 4b) and *SOX9* (Fig. 4c) were similarly expressed in the SMAD2-shRNA and Ctrl-shRNA condition, except for a significant decrease in *COL2A1* expression at day 1 in SMAD2-shRNA (p = 0.007). Although *ACAN* and *SOX9* expression were mildly affected by SMAD3-shRNA, transcription of *COL2A1* was significantly inhibited by SMAD3-shRNA at day 1 (p = 0.007), day 7 (p < 0.001) and day 14 (p = 0.029). Furthermore, a strong and significant inhibition of *ACAN, COL2A1* and *SOX9* expression was observed in SMAD4-shRNA at all time points (p < 0.01 for all genes and time points).

Next, we analyzed cartilage matrix deposition by determining the presence of glycosaminoglycans (GAGs) and collagen type II protein. In addition, we measured the pellet size. Compared to control, TGFβ-induced deposition of GAGs was not inhibited by SMAD2-shRNA at day 14 (Fig. 4d), whereas it was significantly inhibited by SMAD3-shRNA (p = 0.010 at day 7; p < 0.001 at day 14). The same was observed after correcting the GAG content for the amount of DNA per pellet (Fig. 4e; Supplementary Fig. S1). Despite stimulation with TGFβ, no GAGs were formed in pellets of BMSCs transduced with SMAD4-shRNA (Fig. 4d,e). Consistent with this, analyzing the presence of GAGs and collagen type II protein in 14 days-cultured pellets by histology revealed that both cartilage components were similarly present in SMAD2-shRNA and Ctrl-shRNA, whereas they were reduced in SMAD3-shRNA and even absent in SMAD4-shRNA (Fig. 4f). These observations were reflected by the size of the pellets after 14 days of culturing. Compared to Ctrl-shRNA pellets, SMAD2-shRNA pellets had the same size, SMAD3-shRNA pellets were smaller and SMAD4-shRNA pellets were smallest (Fig. 4g).

Once BMSCs have differentiated into chondrocytes, they undergo hypertrophic maturation, which is characterized by enhanced expression of *collagen type X α1 (COL10A1)* and *runt-related transcription factor 2 (RUNX2)*^4^,^5^,^6^,^7^,^8^. We observed that *COL10A1* and *RUNX2* mRNA levels were similar between SMAD2-shRNA, SMAD3-shRNA and Ctrl-shRNA in 7 and 14 days-cultured pellets (Supplementary Fig. S2a,b). In contrast, compared to Ctrl-shRNA, *COL10A1* expression was lower in the SMAD4-shRNA than in the Ctrl-shRNA condition (Supplementary Fig. S2a), while SMAD4-shRNA had no significant effect on *RUNX2* expression (Supplementary Fig. S2b).

Altogether, these results indicate that shRNA-mediated knockdown of SMAD2 had a minor inhibitory effect on TGFβ-induced chondrogenic differentiation of human BMSCs. However, SMAD3 knockdown strongly reduced cartilage deposition, and SMAD4 knockdown completely blocked chondrogenesis.

### SMAD3 overexpression results in a strong inhibition of chondrogenesis

Since knockdown of SMAD2, SMAD3 and SMAD4 inhibited chondrogenesis, we investigated whether overexpression of these SMADs might enhance TGFβ-induced chondrogenic differentiation of human BMSCs.

**Figure 5:**
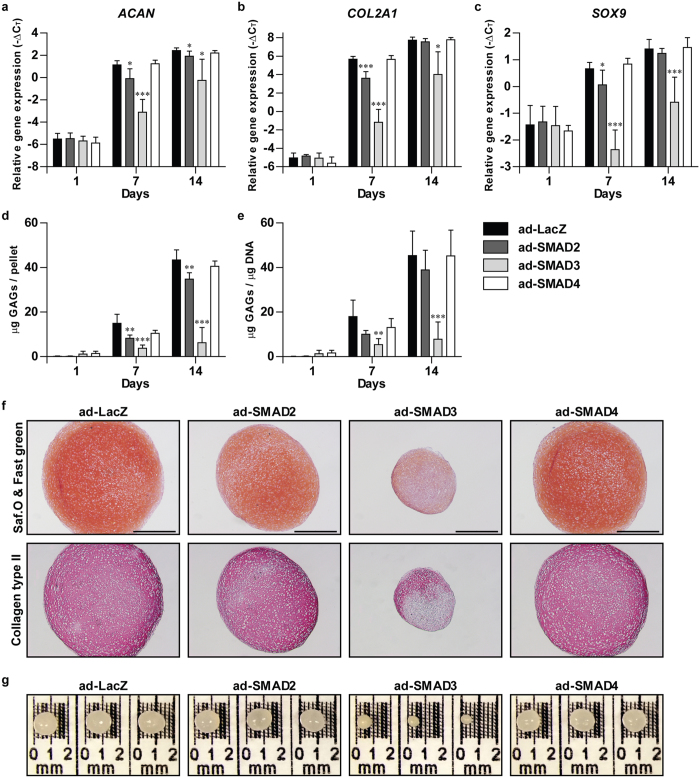
Chondrogenesis is strongly inhibited by SMAD3 overexpression, whereas it is mildly inhibited by SMAD2 overexpression and unaffected by SMAD4 overexpression. Human fetal BMSCs transduced either with adenoviral SMAD2 (ad-SMAD2), ad-SMAD3, ad-SMAD4 or ad-LacZ as control were pellet-cultured for 1, 7 or 14 days in chondrogenic medium with TGFβ. The effect of SMAD overexpression on chondrogenesis was determined by RT-qPCR analysis of the chondrogenesis-specific genes; *ACAN* (**a**), *COL2A1* (**b**) and *SOX9* (**c**), deposition of glycosaminoglycans (GAGs) per pellet (**d**) and per μg DNA (**e**), histological examination of GAGs by Safranin O (Saf.O) staining and collagen type II by immunohistochemistry in sections of 14 days-cultured pellets (**f**), and macroscopic evaluation of 14 days-cultured pellets (**g**). In (**f**) representative images of consecutive pellet sections per condition are shown and the scale bar represents 500 μm. Gene expression data are presented as −ΔC_T_ compared to the mean C_T_ value of *RPS27a* and *TBP*. Bars represent mean + S.D. of triplicate pellets from 2 experiments. *p < 0.05; **p < 0.01; ***p < 0.001 compared to ad-LacZ.

Expression of *ACAN* (Fig. 5a), *COL2A1* (Fig. 5b) and *SOX9* (Fig. 5c) was comparable between the control condition (ad-LacZ) and ad-SMAD2, ad-SMAD3 and ad-SMAD4 after 1 day of pellet culturing. In 7 days-cultured pellets *ACAN, COL2A1* and *SOX9* were slightly lower expressed in ad-SMAD2 than in ad-LacZ (*ACAN*: p = 0.016; *COL2A1*: p < 0.001; *SOX9*: p = 0.047). However, after 14 days only *ACAN* expression was significantly decreased in ad-SMAD2 compared to ad-LacZ (p = 0.038). At all time points expression of *ACAN, COL2A1* and *SOX9* was significantly reduced by ad-SMAD3 (p < 0.05 for all genes), whereas their expression was not affected by ad-SMAD4.

Deposition of GAGs (Fig. 5d) was significantly decreased in pellets of BMSCs transduced with ad-SMAD2 compared to control after 7 days (p = 0.009) and 14 days (p = 0.003). However, this significant reduction was not observed when the GAG content was corrected for the DNA content (Fig. 5e; Supplementary Fig. S3). Pellets of ad-SMAD3-transduced BMSCs had a significantly lower amount of GAGs per pellet (Fig. 5d: p < 0.001 at day 7; p = 0.006 at day 14) and per μg DNA (Fig. 5e; Supplementary Fig. S3; p = 0.007 at day 7; p < 0.001 at day 14) than the control condition. At all time points, GAG deposition (Fig. 5d,e) was similar between ad-SMAD4 and ad-LacZ. Histological analysis of 14 days-cultured pellets revealed that the abundance of GAGs and collagen type II protein (Fig. 5f) was similar between ad-SMAD2, ad-SMAD4 and ad-LacZ, whereas their abundance was strongly reduced in the ad-SMAD3 condition. In line with these observations, pellets of ad-SMAD2-transduced BMSCs were slightly smaller than control pellets, whereas pellets of ad-SMAD3-transduced BMSCs were much smaller (Fig. 5g). The pellet size was comparable between the ad-SMAD4 and ad-LacZ condition (Fig. 5g).

In addition, we found that chondrocyte hypertrophy markers, *COL10A1* and *RUNX2,* were similarly expressed between ad-LacZ and ad-SMAD4 after 7 and 14 days (Supplementary Fig. S4a,b). Although *COL10A1* expression was lower in ad-SMAD2 than in ad-LacZ at day 7, the two conditions were comparable at day 14 (Supplementary Fig. S4a). At both time points, *RUNX2* mRNA levels were similar between ad-SMAD2 and ad-LacZ (Supplementary Fig. S4b). Strikingly, in the ad-SMAD3 condition *COL10A1* mRNA was lower (Supplementary Fig. S4a), whereas *RUNX2* mRNA was higher than in the ad-LacZ condition (Supplementary Fig. S4b).

Our data demonstrate that TGFβ-induced chondrogenesis was slightly inhibited by SMAD2 overexpression and not affected by SMAD4 overexpression, whereas it was strongly inhibited by SMAD3 overexpression.

## Discussion

The pro-chondrogenic effect of TGFβ on human BMSCs is well-known, however, the contribution of the downstream signaling molecules SMAD2, SMAD3 and SMAD4 during chondrogenesis has been less well investigated. By using human fetal BMSCs as a model for TGFβ-induced chondrogenic differentiation, we demonstrate here that modulating SMAD2 expression had a minor effect on chondrogenesis. In contrast, knockdown as well as overexpression of SMAD3 strongly inhibited cartilage formation and SMAD4 knockdown completely blocked chondrogenesis.

To the best of our knowledge, the specific role of SMAD2 and SMAD3 during chondrogenic differentiation of human BMSCs has been studied in one study so far, which demonstrated that modulating SMAD2 expression does not affect chondrogenesis^22^. In contrast, we observed a slight reduction in cartilage formation when SMAD2 was knocked down or overexpressed. We also found that SMAD2 knockdown led to reduced protein levels of SMAD3 and SMAD4, whereas Furumatsu *et al*. did not report an effect of SMAD2 knockdown on SMAD3 and SMAD4 expression^22^. Since knockdown of either SMAD3 or SMAD4 inhibited chondrogenesis, reduced SMAD3 and SMAD4 abundance might explain why chondrogenesis was slightly inhibited by SMAD2 knockdown. Our finding that TGFβ-induced chondrogenic differentiation of human BMSCs is more strongly inhibited by SMAD3 knockdown than by SMAD2 knockdown is in line with the aforementioned study^22^. While we employed continuous knockdown by viral transduction with shRNA, Furumatsu and co-workers employed transient knockdown by transfection with siRNA^22^. We, thus, verified that continuous knockdown of SMAD2 and SMAD3 had effects on chondrogenesis similar to transient knockdown.

Whereas the previous study has shown that SMAD3 overexpression accelerated chondrogenesis^22^, in our study SMAD3 overexpression blocked chondrogenic differentiation of human BMSCs. This discrepancy might be caused by a difference in chondrogenic media composition (details of medium components were not reported), cell source (fetal or adult origin, bone marrow or other tissue derived) or amount of overexpression (western blots of overexpressed SMAD3 were not shown). In addition, we found that the DNA content per pellet decreased over time in cells overexpressing SMAD3 (Supplementary Fig. S3). In multiple cell types, it has been shown that overexpression of SMAD3 leads to an inhibition of proliferation^41^,^42^,^43^. Importantly, proliferation of BMSCs early during chondrogenic differentiation is required for chondrogenesis^44^. Therefore, SMAD3 overexpression might have inhibited chondrogenesis, because it blocked proliferation. Overall, this indicates that constantly high levels of SMAD3 in BMSCs do not accelerate cartilage matrix formation. Since both knockdown and overexpression of SMAD3 inhibited chondrogenic differentiation, chondrogenesis seems to rely on well-balanced levels of SMAD3.

The present study, for the first time, investigated the role of SMAD4 during chondrogenic differentiation of human BMSCs. Previous studies in mice have demonstrated that mesenchyme-specific deletion of *Smad4* leads to an absence of the limb skeleton as a result of impaired mesenchymal condensation^30^,^31^; a process required for initiating chondrogenesis^45^. In our study, SMAD4 knockdown did not interfere with pellet formation, implying that human BMSCs can form pellets even with a low amount of SMAD4. Although BMSCs transduced with SMAD4-shRNA formed pellets, they did not undergo chondrogenic differentiation. This observation is in line with a previous study demonstrating that BMSCs isolated from *Smad4* knockout mice show reduced expression of *Sox9*^30^. Additionally, we found that SMAD4 knockdown inhibited TGFβ-induced SMAD1/5/9 phosphorylation and expression of *COL2A1;* a direct transcriptional target of SOX9^46^. In murine chondroprogenitor cells, complex formation between Smad4 and phosphorylated Smad1/5 proteins is required for transactivation of the *Col2a1* promoter^47^. This might explain why chondrogenesis did not occur when we knocked down SMAD4. Furthermore, we previously demonstrated that the SMAD2/3 as well as the SMAD1/5/9 pathways are required for TGFβ-induced chondrogenesis of human BMSCs^6^,^10^. In the present study activation of both pathways was reduced by SMAD4 knockdown, thereby possibly explaining the absence of cartilage formation. Altogether, these findings underline the importance of SMAD4 during TGFβ-induced chondrogenic differentiation of human BMSCs.

Following chondrogenesis, BMSC-derived chondrocytes display signs of hypertrophic differentiation. This is undesired for the formation of articular cartilage, as hypertrophic chondrocytes produce cartilage that will mineralize and ossify when implanted *in vivo*^7^. We found that modulating SMAD2 expression did not have an effect on expression of hypertrophic differentiation markers, neither did SMAD4 overexpression. SMAD3 overexpression and SMAD4 knockdown did result in lower *COL10A1* expression. In these conditions, however, next to hypertrophy markers, the markers of chondrogenesis were also much lower expressed than in the control condition, following the principle that reduced chondrogenesis leads to reduced hypertrophic differentiation. Whereas *COL10A1* expression was lower by SMAD3 overexpression, *RUNX2* expression was slightly, but significantly, higher. This was surprising, because SMAD3 is required for repression of *RUNX2* expression and chondrocyte hypertrophy^38^,^48^,^49^,^50^,^51^. Possibly, as a result of decreased chondrogenesis *RUNX2* expression failed to go down during the early phase of chondrogenesis or *RUNX2* expression levels might depend on the abundance of SMAD3, but this requires further investigation. Overall, our results failed to show effects specifically on hypertrophic differentiation of BMSC-derived chondrocytes without effects on the induction of chondrogenesis.

Besides the effects on chondrogenic and hypertrophic differentiation, this study investigated the effect of modulating SMAD2, SMAD3 and SMAD4 expression on TGFβ signal transduction. After activation by TGFβ, the ALK5 receptor phosphorylates SMAD2/3 proteins^9^. We show that SMAD2 phosphorylation was reduced by knockdown of SMAD2, while it highly increased by SMAD2 overexpression. These findings suggests that TGFβ-activated phosphorylation depends on the number of R-SMADs present in the cytoplasm rather than, for instance, on the kinase activity or number of ALK5 receptors.

We previously showed that TGFβ does not only induce phosphorylation of SMAD2/3, but also of SMAD1/5/9 in BMSCs^6^,^10^,^52^. Although SMAD1/5/9 cannot be directly phosphorylated by the TGFβ receptor ALK5, TGFβ-activated SMAD1/5/9 phosphorylation requires the intracellular kinase domains of ALK5^40^. Moreover, ALK5 is the only receptor through which TGFβ can induce phosphorylation of SMAD2/3^53^,^54^. Consistent with a study showing enhanced pSMAD1/5/9 in SMAD3 knockout chondrocytes^51^, we demonstrate increased pSMAD1/5/9 when SMAD3 or SMAD2 were knocked down. This supports the idea that TGFβ-induced phosphorylation of SMAD1/5/9 requires the ALK5 receptor and is independent of other receptors that can activate SMAD1/5/9^55^,^56^.

Although SMAD4 is an important co-factor for translocation of activated R-SMADs to the nucleus^57^, it has not been implicated in controlling the phosphorylation of R-SMADs. Patients with Myhre syndrome have mutations in *SMAD4* that lead to decreased ubiquitination of SMAD4 protein, resulting in accumulation of SMAD4^58^,^59^,^60^,^61^. In skin fibroblasts from these patients, pSMAD2/3 and pSMAD1/5/9 were enhanced compared to healthy controls^58^,^61^. Contrary to this, in our study SMAD4 overexpression had no effect on R-SMAD phosphorylation. On the other hand, SMAD4 knockdown reduced TGFβ-induced phosphorylation of SMAD2 and completely prevented SMAD1/5/9 phosphorylation. Based on these observations, we speculate that SMAD4 is required for R-SMAD phosphorylation, or prevents R-SMADs from de-phosphorylation^62^,^63^ or ubiquitination^64^,^65^, which adds a regulatory mechanism that controls TGFβ signal transduction.

To conclude, this study reveals that TGFβ-activated phosphorylation of R-SMADs in BMSCs does not only depend on the levels of SMAD2 and SMAD3, but also on the presence of SMAD4. Moreover, our findings suggest that SMAD3 and SMAD4 are more important than SMAD2 for TGFβ-induced chondrogenic differentiation of human BMSCs. However, as cartilage formation was not enhanced by overexpression of SMAD4 and even inhibited by SMAD3 overexpression, induction of chondrogenic differentiation seems to rely on a delicate balance in the amount of SMAD3 and SMAD4. This also implies that continuously enhanced SMAD3 expression levels may not be a suitable strategy to improve chondrogenesis. Despite its exploratory nature, this study offers novel insights into the signaling mechanism governing the induction of chondrogenic differentiation of human BMSCs. Further research is required to delineate the function of SMAD4 in TGFβ-induced phosphorylation of R-SMADs and the mechanisms behind the detrimental effects of SMAD3 overexpression on chondrogenesis.

## Methods

### Culturing of human fetal bone marrow-derived mesenchymal stem cells

Human fetal BMSCs (#SCC7500, Lot#6890, ScienCell Research Laboratories, Carlsbad, CA, USA) were expanded in Mesenchymal Stem Cell Growth Medium (MSCGM™; Lonza, Basel, Switzerland) supplemented with 1% Penicillin-Streptomycin-Glutamine (Gibco, Carlsbad, CA, USA). Cells were cultured in a 37 °C-incubator with 5% CO_2_. After reaching 80% confluence, cells were passaged using 0.05% trypsin-EDTA (Gibco) and re-seeded (~6,000 cells/cm^2^) in MSCGM. After 4 passages, cells were stored in liquid nitrogen. Per experiment, fetal BMSCs were defrosted and expanded in MSCGM for another 2 or 3 passages.

### Induction of chondrogenic differentiation

BMSC pellets were obtained by centrifuging 200,000 cells at 300 × g for 8 minutes in polystyrene V-bottom tubes (Greiner Bio-One, Alphen a/d Rijn, Netherlands). Pellets were cultured for 1, 7 or 14 days in 0.5 mL of serum-free chondrogenic medium, which consisted of DMEM-high glucose-GlutaMAX, 1% Penicillin-Streptomycin-Glutamine (both from Gibco), 6.25 μg/mL Insulin, 6.25 μg/mL Transferrin, 6.25 ng/mL selenious acid 5.35 μg/mL linoleic acid, 1.25 mg/mL bovine serum albumin, 1.0 mg/mL sodium pyruvate, 0.4 mg/mL L-proline, 50 μg/mL sodium L-ascorbate, 10^−7^ M dexamethasone (all from Sigma-Aldrich), and 10 ng/mL TGFβ1 (Biolegend, San Diego, CA, USA). This medium was renewed 3 times per week.

### Short hairpin-mediated knockdown of SMAD2, SMAD3 or SMAD4

MISSION^®^ TRC-Hs1.5 shRNA clones targeting SMAD2 (TRCN0000040036), SMAD3 (TRCN0000330056) or SMAD4 (TRCN0000010321), and Non-Mammalian shRNA control (SHC002) constructed in the pLKO.1-Puro plasmid vector were obtained from Sigma-Aldrich. Lentiviruses were packaged as described previously^10^. Briefly, HEK293T cells (ATCC, Manassas, VA, USA) were co-transduced with plasmids of Rev, Gag, Pol, VSV-G (Plasmid Factory, Bielefeld, Germany) and a pLKO.1-Puro plasmid vector by calcium phosphate precipitation in DMEM (Gibco) containing 10% FCS (Perbio Science, Erembodegem, Belgium), 0.01 mM cholesterol (Sigma-Aldrich) and 1% pyruvate (Gibco). Medium was renewed at day 1, 2 and 3 post-transduction, collecting the medium at day 2 and 3. Collected medium was filtered through a 0.45 μm filter and centrifuged at 134,350 × g for 2 hours (Sorvall WX80+, ThermoFisher Scientific). Lentivirus concentration was determined with the INNOTEST^®^ HIV p24 Antigen assay (Fujirebio Europe, Gent, Belgium) and expressed as pg of p24/μL.

At 20% confluence, BMSCs were infected with 1 pg p24 per cell in MSCGM (Lonza) supplemented with 100 μg/mL protamine sulfate (Sigma-Aldrich) for 1 day. After culture-expanding infected cells for 2 days, pellets were prepared to induce chondrogenesis. 1 day after pellet preparation, the efficiency of shRNA-mediated knockdown of SMAD2, SMAD3 and SMAD4 was analyzed by RT-qPCR and Western blot.

### Adenoviral-mediated overexpression of SMAD2, SMAD3 or SMAD4

Adenoviruses for SMAD2, SMAD3 and SMAD4 were kindly provided by Dr. P. ten Dijke (Leiden University Medical Center, the Netherlands). Cells at 80% confluence were incubated for 3 hours with adenovirus (multiplicity of infection of 1 pfu/cell) overexpressing either SMAD2, SMAD3, SMAD4 or LacZ (control). Following transduction, cells were washed and centrifuged to obtain pellets for chondrogenic induction. Overexpression of SMAD2, SMAD3 and SMAD4 was verified at gene (RT-qPCR) and protein (Western blot) level in BMSCs cultured for 1 day in chondrogenic medium.

### Western blot analysis of (phosphorylated) SMAD proteins

To evaluate the effects of SMAD knockdown and overexpression on activation of TGFβ signaling, cells transduced either with lentivirus (SMAD-shRNA) or adenovirus (SMAD overexpression) were seeded in chondrogenic medium without TGFβ. After 18 hours, cells were stimulated with 10 ng/mL TGFβ1 (Biolegend) for 1 hour and cell lysates were prepared as described below to determine pSMAD2 and pSMAD1/5/9 expression.

To determine protein abundance, cells were lysed (duplicate per condition) using lysis buffer (Cell-Signaling-Technology, Danvers, MA, USA) containing 1% protease inhibitor (Roche, Mannheim, Germany) and lysates were sonicated on ice. Protein concentration was determined using bicinchoninic acid assay. Duplicate samples per condition were pooled and 10 μg protein lysate was subjected to 10% sodium dodecyl sulfate-polyacrylamide gel electrophoresis. Proteins were transferred to nitrocellulose membranes and overnight at 4 °C incubated with an antibody (1:1,000) recognizing SMAD2 (#40855, Abcam, Cambridge, UK), SMAD3 (#28379, Abcam), SMAD4 (#AF2097, R&D systems, Minneapolis, MN, USA), pSMAD2 (#3101 L, Cell Signaling Technology) or pSMAD1/5/9 (#9511 L, Cell Signaling Technology). Subsequently, membranes were incubated with HRP-linked antibody (1:1,500) against rabbit-IgG (#P0448, Dako, Glostrup, Denmark) or against goat-IgG (#P0449, Dako) for 1 hour at room temperature. To evaluate equal protein loading between conditions, GAPDH expression was determined. Membranes were overnight incubated with an antibody (1:20,000) recognizing GAPDH (#G8795, Sigma-Aldrich), followed by 1 hour-incubation with HRP-linked antibody (1:1,500) against mouse-IgG (#P0260, Dako). Proteins were visualized by enhanced chemiluminescence using Prime Western Blotting Detection Reagent and a ImageQuant LAS4000 machine (GE Healthcare). Densitometry was performed using ImageJ software (release 1.46r; National Institute of Health, Bethesda, Maryland, USA).

### RNA extraction and gene expression analysis

Pellets (n = 3 per condition at each time point) were collected in TRIzol^®^ (Sigma-Aldrich) and disrupted using MagNA Lyser instrument (Roche). After total RNA was isolated according to manufacturer’s protocol (Sigma-Aldrich), RNA samples were treated with DNAse (Invitrogen) to remove contaminating genomic DNA. RNA concentration and purity were measured using a NanoDrop^®^ spectrophotometer (Isogen Life Science, Utrecht, the Netherlands). 0.5 μg RNA was reverse transcribed in cDNA with M-MLV Reverse Transcriptase (Invitrogen). Gene expression was measured by real-time Reverse Transcription Quantitative Polymerase Chain Reaction (RT-qPCR) on a StepOnePlus™ System using SYBR Green Master mix (Applied Biosystems) and the primers listed in Table 1. C_T_ values were determined at a fixed threshold level of fluorescence and efficiency of all primers was between 90% and 110% (Table 1). Data were normalized to the mean C_T_ value of *Ribosomal protein 27a (RPS27A)* and *TATA-box binding protein (TBP)*. The following genes were used as markers of chondrogenesis; *Aggrecan (ACAN), Collagen type IIα1 (COL2A1)* and *SRY (Sex Determining Region Y)-Box 9 (SOX9)*. In addition, markers of chondrocyte hypertrophy; *collagen type 10 α1 (COL10A1)* and *runt-related transcription factor 2 (RUNX2)*, were measured.

### Histology

To macroscopically evaluate pellet size of 14-days cultured pellets, pictures were taken using a microscope (Wild M3B, Heerbrugg, Switzerland). Subsequently, pellets were fixed in 4% formalin for 14 hours, embedded in paraffin and sectioned (6 μm). Sections were stained with 0.1% aqueous Safranin O (Brunschwig Chemie, Amsterdam, the Netherlands), resulting in red-staining of negatively-charged GAGs. As counter-staining, 0.1% aqueous Fast Green (Brunschwig Chemie) was used to stain the cytoplasm blue/green.

For immunohistochemical staining of collagen type II, sections were pre-treated with 1 mg/mL pronase and 10 mg/mL hyaluronidase (Sigma-Aldrich), and then incubated with 0.4 μg/mL antibody specific for collagen type II (#II-II6B3, Developmental Studies Hybridoma Bank, Iowa City, IA, USA) or 0.4 μg/mL mouse-IgG1 (#X0931, Dako, Glostrup, Denmark). Following incubation with alkaline phosphatase (AP)-conjugated secondary antibody (1:50, #HK-321-UK, Biogenex, San Ramon, CA, USA), AP-activity was visualized (magenta color) by incubation with new-fuchsin substrate. Sections were counterstained with haematoxylin (purple) to visualize nuclei.

### Glycosaminoglycans and DNA content measurements

Pellets cultured for 1, 7 or 14 days were digested overnight at 60 °C in 100 μL papain digestion buffer (pH = 6.4) containing 0.1% papain, 10 mM EDTA-disodium salt (both from Merck), 100 mM sodium acetate, and 5 mM L-Cysteine·HCL (both from Sigma-Aldrich). Following digestion, samples were diluted 1:8 in water to measure the GAG content or samples were diluted 1:6 in Tris-EDTA (TE) buffer to measure DNA content. The GAG content was measured by adding 200 μL dimethylmethylene blue (DMB) solution (0.05 mM DMB, 41 mM NaCl, 45 mM glycin and pH = 3.0) to 40 μL papain-digested sample (pre-diluted 1:8 in water) and absorbance was measured at 590 nm using an iMark Reader (Bio-Rad). To determine the DNA content, PicoGreen^®^ stock solution (ThermoFisher Scientific) was diluted 1:200 in TE buffer (10 mM Tris-HCl, 1 mM EDTA and pH = 7.5) and 50 μL of this solution was mixed with 50 μL papain-digested sample (pre-diluted 1:6 in TE buffer). After 5 minutes dark incubation at room temperature, fluorescence was measured at 485/520 nm (excitation/emission) with a CLARIOstar (BMG Labtech, Offenburg, Germany) using DNA obtained from human HEK293T cells to set the standard curve.

### Data analysis

Data represent mean + standard deviation of 6 pellets (from two experiments with triplicate pellets per experiment) per condition per time point. Statistical analyses were performed using SPSS version 22 (IBM, Armonk, NY, USA). Normality and variance were verified with the Shapiro-Wilk test and Levene’s Test, respectively. Statistical differences between control and experimental conditions per time point were determined with two-tailed independent T-tests. Level of significance was set at P < 0.05.

## Additional Information

**How to cite this article**: de Kroon, L. M. G. *et al*. SMAD3 and SMAD4 have a more dominant role than SMAD2 in TGFβ-induced chondrogenic differentiation of bone marrow-derived mesenchymal stem cells. *Sci. Rep.* **7**, 43164; doi: 10.1038/srep43164 (2017).

**Publisher's note:** Springer Nature remains neutral with regard to jurisdictional claims in published maps and institutional affiliations.

## Supplementary Material

Supplementary Figures

## Figures and Tables

**Table 1:** List of primers used for RT-qPCR.

Gene	Forward primer	Reverse primer	Product
*RPS27A*	TGGCTGTCCTGAAATATTATAAGGT	CCCCAGCACCACATTCATCA	90 bp
*TBP*	GCTTCGGAGAGTTCTGGGATTG	GCAGCAAACCGCTTGGGATTA	134 bp
*SMAD2*	CCGACACACCGAGATCCTAAC	AGGAGGTGGCGTTTCTGGAAT	127 bp
*SMAD3*	CATCGAGCCCCAGAGCAATA	GTGGTTCATCTGGTGGTCACT	88 bp
*SMAD4*	CCAATCATCCTGCTCCTGAGT	CCAGAAGGGTCCACGTATCC	130 bp
*ACAN*	GCCTGCGCTCCAATGACT	ATGGAACACGATGCCTTTCAC	104 bp
*COL2A1*	CACGTACACTGCCCTGAAGGA	CGATAACAGTCTTGCCCCACTT	65 bp
*SOX9*	TGGGCAAGCTCTGGAGACTT	CCCGTTCTTCACCGACTTCCT	140 bp
*COL10A1*	TTTTACGCTGAACGATACCAAATG	CTGTGTCTTGGTGTTGGGTAGTG	66 bp
*RUNX2*	GCAAGGTTCAACGATCTGAGA	TTCCCGAGGTCCATCTACTG	141 bp
