# Supplementary material for: SMAD3 and SMAD4 have a more dominant role than SMAD2 in TGFβ-induced chondrogenic differentiation of bone marrow-derived mesenchymal stem cells
Source: Sci Rep. 2017 Feb 27;7:43164. doi: 10.1038/srep43164 (PMC5327413; doi:10.1038/srep43164)
Supplement: Supplementary Figures [file srep43164-s1.pdf]

SMAD3 and SMAD4 have a more dominant role than SMAD2 in TGFβ-induced chondrogenic differentiation of bone marrow-derived mesenchymal stem cells

Laurie M. G. de Kroon[1,2], Roberto Narcisi[2], Guus G. H. van den Akker[1], Elly L. Vitters[1], Esmeralda N. Blaney Davidson[1], Gerjo J. V. M. van Osch[2,3], and Peter M. van der Kraan[1,*]

[1]Experimental Rheumatology, Department of Rheumatology, Radboud University Medical Center, Nijmegen, 6500 HB, the Netherlands
[2]Department of Orthopedics, Erasmus MC University Medical Center, Rotterdam, 3015 CN, the Netherlands
[3]Department of Otorhinolaryngology, Erasmus MC University Medical Center, Rotterdam, 3015 CN, the Netherlands
[*]Peter.vanderKraan@radboudumc.nl

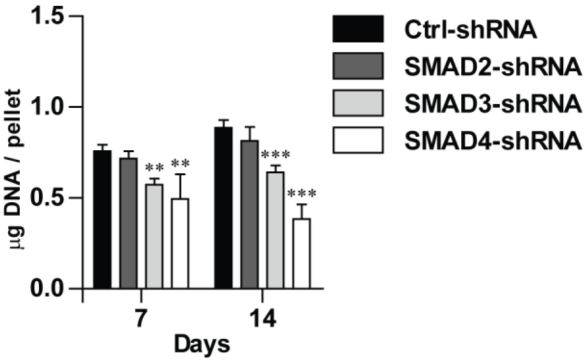

Supplementary Figure S1. The DNA content per pellet is lower in SMAD3-shRNA and SMAD4-shRNA than in Ctrl-shRNA. Human fetal BMSCs were transduced (lentivirus) either with SMAD2-shRNA, SMAD3-shRNA, SMAD4-shRNA or Ctrl-shRNA. Subsequently, BMSCs were pellet-cultured for 7 and 14 days in chondrogenic medium with TGFβ. The DNA content per pellet was measured using Picogreen. Bars represent mean + S.D. of triplicate pellets from 2 experiments. **=$p<0.01$; ***=$p<0.001$ compared to Ctrl-shRNA.

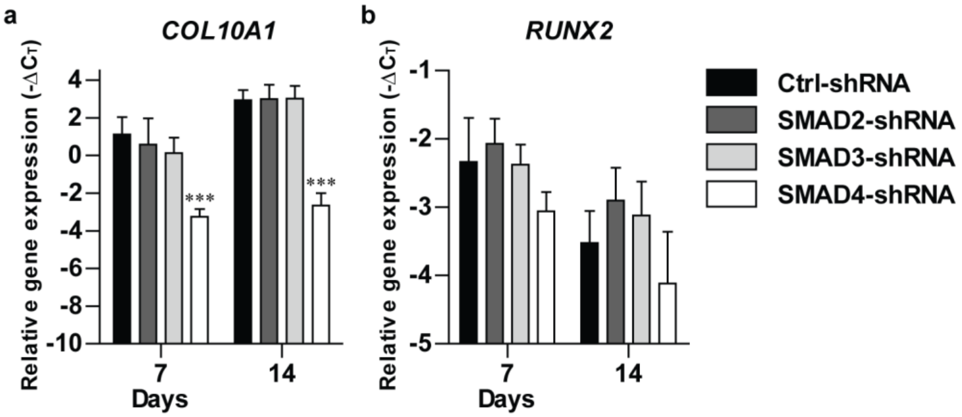

Supplementary Figure S2. Effect of knockdown of SMAD2, SMAD3 or SMAD4 on chondrocyte hypertrophy markers. The effect of shRNA-mediated knockdown of SMAD2, SMAD3 and SMAD4 on gene expression of markers for hypertrophic differentiation of chondrocytes; *collagen type 10 α1 (COL10A1)* **(a)** and *runt-related transcription factor 2 (RUNX2)* **(b)**, was measured by RT-qPCR in 7 and 14 days-cultured human fetal BMSC pellets. Gene expression data are presented as $-\Delta C_T$ compared to the mean C_T value of *RPS27a* and *TBP*. Bars represent mean + S.D. of triplicate pellets from 2 experiments. ***=p<0.001 compared to Ctrl-shRNA.

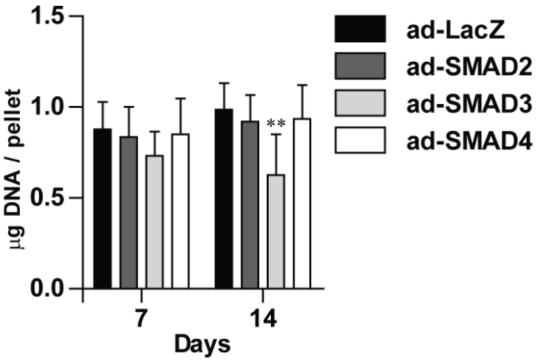

Supplementary Figure S3. The DNA content per pellet is reduced by SMAD3 overexpression. Human fetal BMSCs were transduced either with ad-SMAD2, ad-SMAD3, ad-SMAD4 or ad-LacZ as control. Subsequently, BMSCs were pellet-cultured for 7 and 14 days in chondrogenic medium with TGFβ. The DNA content per pellet was measured using Picogreen. Bars represent mean + S.D. of triplicate pellets from 2 experiments. **=$p<0.01$ compared to ad-LacZ.

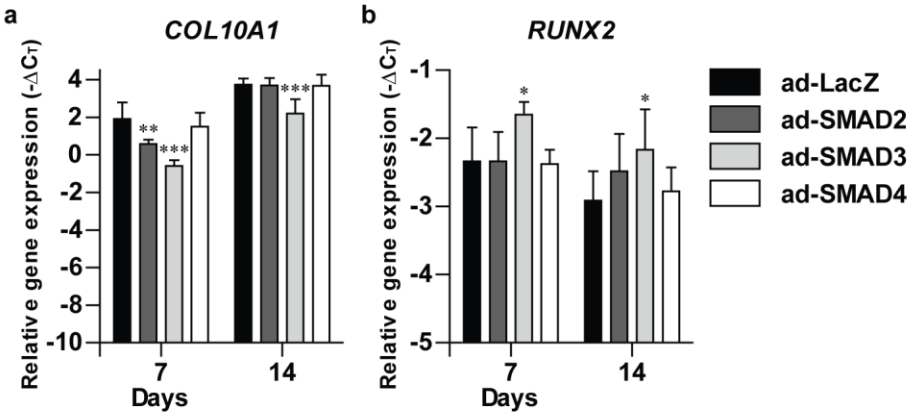

Supplementary Figure S4. Effect of overexpression of SMAD2, SMAD3 or SMAD4 on chondrocyte hypertrophy markers. The effect of adenoviral (ad)-mediated overexpression of SMAD2, SMAD3 and SMAD4 on gene expression of markers for hypertrophic differentiation of chondrocytes; *collagen type 10 α1 (COL10A1)* **(a)** and *runt-related transcription factor 2 (RUNX2)* **(b)**, was measured by RT-qPCR in 7 and 14 days-cultured human fetal BMSC pellets. Gene expression data are presented as $-\Delta C_T$ compared to the mean C_T value of *RPS27a* and *TBP*. Bars represent mean + S.D. of triplicate pellets from 2 experiments. *=p<0.05; **=p<0.01; ***=p<0.001 compared to ad-LacZ.
